# Barriers to Receipt of Prenatal Tetanus Toxoid, Reduced Diphtheria Toxoid, and Acellular Pertussis Vaccine Among Mothers of Infants Aged <4 Months with Pertussis — California, 2016

**DOI:** 10.15585/mmwr.mm6738a6

**Published:** 2018-09-28

**Authors:** Sarah New, Kathleen Winter, Rebeca Boyte, Kathleen Harriman, Anya Gutman, Amber Christiansen, Sarah Royce

**Affiliations:** ^1^Immunization Branch, California Department of Public Health; ^2^Department of Epidemiology, University of Kentucky, Lexington, Kentucky.

Vaccination with tetanus toxoid, reduced diphtheria toxoid, and acellular pertussis (Tdap) vaccine is recommended for all pregnant women to protect infants who are too young for vaccination from severe pertussis-related outcomes (*1*–*3*). However, Tdap vaccine coverage among pregnant women remains suboptimal in California (*4*). California mothers whose infants developed pertussis in 2016 and their prenatal care providers were interviewed to ascertain possible reasons for low Tdap vaccine coverage. Mothers who were offered Tdap vaccination on-site during a routine prenatal visit were more likely to be vaccinated than were mothers who were referred off-site for vaccination. Mothers insured by Medicaid were less likely to receive Tdap vaccine than were mothers with private insurance, even when the vaccine was stocked on-site. Nearly all vaccinated mothers received Tdap vaccine in their prenatal clinic. Incorporating Tdap vaccination into routine prenatal care visits is an effective means to increase prenatal Tdap vaccination coverage.

Most severe and fatal cases of pertussis occur in infants who have not yet started the primary pertussis vaccination series. To reduce the incidence of pertussis in these young infants, the Advisory Committee on Immunization Practices (ACIP) recommends that pregnant women receive Tdap vaccine at the earliest opportunity, during 27–36 weeks’ gestation of each pregnancy (*3*). Vaccination during this period results in optimal transplacental transfer of maternal pertussis antibodies, which provides infants with passive protection against pertussis during the first weeks of life (*1*–*3*). Despite this recommendation, prenatal Tdap vaccine coverage in California remains low, with 52% of pregnant women estimated to have been vaccinated, and coverage is even lower (40%) among women with Medicaid insurance (*5*). In 2016, a low-incidence pertussis year in California, 114 pertussis cases, which included two pertussis-related deaths, occurred among infants aged <4 months.

California local health department personnel completed a case report form and attempted to complete a supplemental questionnaire using information collected during routine case investigation interviews with mothers of all 114 infants aged <4 months with pertussis who had illness onset during 2016. Interviewed mothers were asked to identify their prenatal care provider; whether they received a recommendation for Tdap vaccination during pregnancy with the case infant; whether they received Tdap vaccine; and if so, the date and location it was administered. Mothers who reported that they did not receive Tdap vaccine during pregnancy were asked why Tdap vaccine was not administered. Prenatal care providers were asked to identify the mothers’ insurance type during pregnancy; whether Tdap vaccination was recommended during pregnancy; whether Tdap vaccine was stocked in the clinic; and if not, why it was not stocked. Providers were also asked to verify the mothers’ Tdap vaccination status, and if Tdap vaccine was administered, the date and gestational week of administration. If mothers were referred off-site for Tdap vaccination, providers were asked whether they followed up to ensure that Tdap vaccination occurred. In addition, the California Immunization Registry was searched in an attempt to identify doses of Tdap vaccine that were not reported during interviews. However, this registry was mandated in late 2016 for pharmacists only. Additional variables collected from infant pertussis case reports included whether the infant was hospitalized or admitted to an intensive care unit (ICU) and the infant’s outcome. Relative risks and 95% confidence intervals (CIs) were calculated to identify differences between vaccinated and unvaccinated mothers and barriers to receipt of prenatal Tdap vaccine. Because reporting of pertussis cases in infants aged <4 months is mandated in California, the follow-up interviews and data collected for this analysis were considered to be nonresearch data.

Sixty-six (58%) mothers and their prenatal care providers completed the supplemental questionnaire during routine case investigations. Data on mother’s insurance status, stocking status of Tdap vaccine by provider, or mother’s gestational week of pregnancy (if vaccinated) were incomplete for six (9%) mothers and were excluded from relevant calculations. Twenty-six (39%) of the 66 interviewed mothers reported receiving Tdap vaccine during their pregnancy with the case infant; among these, 24 (92%) were vaccinated at their prenatal care provider’s office. Prenatal care providers documented Tdap vaccine administration for 25 of the 26 mothers who reported vaccination; no information on a Tdap vaccine dose was found in the medical record or the California Immunization Registry for one mother. Among the 25 mothers with documentation of receipt of prenatal Tdap vaccine, 20 (80%) were vaccinated according to ACIP recommendations, during 27–36 weeks’ gestation (median = 32 weeks). Five (20%) mothers received Tdap vaccine outside the recommended 27–36 week time frame (one each at 26, 38, and 39 weeks’ gestation and two at 37 weeks). Among the 20 infants whose mothers were vaccinated during the recommended time frame, four were hospitalized, but none required admission to an ICU. Among the five infants whose mothers were vaccinated outside the recommended time frame, two were hospitalized, including one who required ICU admission and subsequently died.

Among the 66 interviewed mothers, 40 (61%) did not receive Tdap vaccine during pregnancy and among these mothers, 20 (50%) of their infants were hospitalized, including eight (40%) who were admitted to an ICU, one of whom died. Among the 40 unvaccinated mothers, 10 (25%) did not receive a recommendation or referral off-site for vaccination from their prenatal care provider, nine (23%) were referred off-site for vaccination but did not receive Tdap vaccine, eight (20%) mothers reported refusing Tdap vaccine for personal reasons, seven (18%) were deferred for vaccination by their provider for a reason not considered by ACIP to be a contraindication (prior receipt of Tdap vaccine, minor illness, or current medication use), three (8%) did not receive prenatal care during 27–36 weeks’ gestation, one (3%) reported a possible valid contraindication (adverse reaction to pertussis vaccination as a child), and no information was available for two (5%) women (*6*) (Table 1). Sixteen (40%) of the 40 mothers who were not vaccinated during pregnancy received Tdap vaccine postpartum (the recommended strategy before 2011).

**TABLE 1:** Reasons prenatal Tdap vaccination was not received during pregnancy among interviewed mothers of infants aged <4 months with pertussis (N = 40) — California, 2016

Reason	No. (%)
No recommendation or referral	10 (25)
Referred off-site, did not follow up	9 (23)
Refused for personal reasons	8 (20)
Invalid contraindication*	7 (18)
No prenatal care in third trimester	3 (8)
Valid contraindication	1 (3)
Unknown	2 (5)

**Abbreviation**: Tdap = tetanus toxoid, reduced diphtheria toxoid, and acellular pertussis vaccine.

* Invalid contraindications included prior receipt of prenatal Tdap (two); minor illness (two); previous illness associated with receipt of influenza vaccine (one); family member up to date with pertussis vaccination (one); and current medication use (one).

Among the 60 (91%) mothers with complete information, 19 (73%) of 26 women with private insurance and 15 (44%) of 34 women insured by Medicaid received prenatal care from a provider who stocked Tdap vaccine on-site (Table 2). Fourteen (54%) mothers with private insurance received Tdap vaccine on time, compared with six (18%) of mothers with Medicaid insurance. Mothers whose providers stocked Tdap vaccine on-site were significantly more likely to have been vaccinated (relative risk [RR] = 3.3; 95% CI = 1.9–5.5) than were mothers whose providers did not stock Tdap vaccine. Mothers insured by Medicaid were significantly less likely than those with private insurance to receive prenatal Tdap vaccine (0.4; 0.2–0.8) or to receive prenatal Tdap vaccine during the appropriate time frame, even when it was stocked on-site (0.5; 0.3–1.1).

**TABLE 2:** Prenatal Tdap vaccination outcomes for interviewed mothers of infants aged <4 months with pertussis (N = 60*), by clinic Tdap vaccine stocking policy and mothers’ insurance coverage — California, 2016

Tdap policy/Insurance coverage	Tdap vaccination status no. (%)	
	Received per ACIP recommendations^†^	Not received on time or at all
Tdap stocked on-site in clinic (n = 34)		
Private insurance (n = 19)	14 (41)	5 (15)
Medicaid (n = 15)	6 (18)	9 (26)
**Tdap not stocked on-site in clinic (n = 26)**		
Private insurance (n = 7)	0 (—)	7 (27)
Medicaid (n = 19)	0 (—)	19 (73)
**Unknown (n = 5)**	0 (—)	5 (100)

**Abbreviations:** ACIP = Advisory Committee on Immunization Practices; Tdap = tetanus toxoid, reduced diphtheria toxoid, and acellular pertussis.

* Six of the 66 mothers interviewed were excluded because data on mother’s insurance status, stocking status of Tdap vaccine by provider, or mothers’ gestational week of pregnancy (if vaccinated) were incomplete.

^†^ Among clinics that stocked Tdap vaccine on-site, 20 mothers received Tdap during 27–36 weeks’ gestation.

Among the 61 interviewed prenatal care providers whose Tdap vaccine stocking policies were known, 34 (56%) stocked Tdap vaccine on-site. Among the 27 (44%) providers who did not stock Tdap vaccine on-site, 17 (63%) recommended Tdap vaccine during pregnancy, 16 of whom referred mothers off-site for vaccination, typically to a pharmacy, local public health department, or the mother’s primary care physician. Two (13%) of the 16 mothers referred off-site were vaccinated, including one who was vaccinated at 38 weeks’ gestation. In only one case was the Tdap dose documented in the mother’s medical record. Among the 27 providers who did not stock Tdap vaccine on-site, the most common reasons cited for not stocking were cost (44%) and reimbursement (41%) issues.

## Discussion

In this review of 66 mothers of infants aged <4 months who became ill with pertussis in 2016, 20 mothers (30%) received Tdap vaccine during the time frame recommended by ACIP, all of whom were vaccinated in their prenatal clinic during a routine visit. Mothers whose providers stocked Tdap on-site were more likely to be vaccinated than were those whose providers did not stock Tdap. This finding is consistent with a previous survey demonstrating that receipt of influenza vaccine by pregnant women was more likely among those whose prenatal care providers offered vaccination on-site (*4*). However, even when prenatal care providers stocked Tdap vaccine on-site, women insured by Medicaid were less likely to receive Tdap vaccination than were women with private insurance. Previous Tdap vaccination coverage estimates among pregnant women in California were also lower among those insured by Medicaid (40%) than among those with private insurance (65%) (*5*). Reasons for this disparity are not known; however, a need exists to reduce financial barriers to stocking and administering Tdap vaccine in prenatal clinics, particularly among those serving Medicaid patients.

To prevent pertussis among young infants, women should receive Tdap vaccination during 27–36 weeks’ gestation during every pregnancy. A recommendation by prenatal care providers for their pregnant patients to receive Tdap is important, particularly among providers who do not stock Tdap vaccine on-site. If Tdap vaccine is not stocked on-site, it is important to provide pregnant patients with specific information* about where they can receive Tdap vaccination and to follow up at subsequent visits to ensure Tdap vaccine is received within the recommended time frame (*7*,*8*). Approximately 40% of the unvaccinated mothers in this analysis never received a recommendation or referral for Tdap vaccine or were deferred for reasons that were inconsistent with current ACIP recommendations, and 40% of the unvaccinated mothers, including one who originally refused Tdap vaccination, received Tdap vaccine postpartum, suggesting that prenatal-provider education about current Tdap vaccine recommendations is needed. Eight (20%) of the 40 mothers who were not vaccinated during pregnancy refused prenatal Tdap.

Fewer infants of mothers who were vaccinated according to recommendations required hospitalization after developing pertussis than did infants of unvaccinated mothers. Although not statistically significant, this difference is consistent with a previous report and highlights the importance of prenatal Tdap vaccination in preventing severe outcomes of pertussis (*9*).

The findings in this report are subject to at least one limitation. The relatively small sample size might affect the generalizability of these findings to the U.S. population of pregnant women and their prenatal care providers.

In California, pharmacists are permitted to provide immunizations, and all routinely recommended adult vaccines are covered by Medicaid when given in a provider’s office or in a pharmacy. Recent state regulations require pharmacists to notify providers of immunizations administered and to enter all doses into the California Immunization Registry, making it possible for providers to know whether vaccine referrals to pharmacies are successful. However, stocking vaccines on-site in prenatal clinics is the best way to ensure that all pregnant women are vaccinated and reduce the incidence of pertussis among infants too young to be vaccinated.

**Summary**

**What is already known about this topic?**

Although tetanus toxoid, reduced diphtheria toxoid, and acellular pertussis (Tdap) vaccination is recommended for all pregnant women during 27–36 weeks’ gestation to prevent infant pertussis, coverage among pregnant women is suboptimal.

**What is added by this report?**

Among 66 interviewed mothers of infants aged <4 months with pertussis, 30% appropriately received Tdap vaccine. Women whose clinics stocked Tdap vaccine were more likely to be vaccinated. Women with Medicaid were less likely to be vaccinated than were those with private insurance, even when treated in clinics that stocked Tdap vaccine.

**What are the implications for public health practice?**

Promoting on-site prenatal vaccination, educating providers about Tdap recommendations, and strengthening off-site referral likely will improve Tdap vaccination coverage during pregnancy.

## Footnotes

* http://eziz.org/resources/pertussis-promo-materials/prenatal-tdap/.
